# Supplementary material for: Comparison of open anterior component and open transversus abdominus release in repair of large subcostal hernias
Source: PLoS One. 2023 Dec 29;18(12):e0288571. doi: 10.1371/journal.pone.0288571 (PMC10756514; doi:10.1371/journal.pone.0288571)
Supplement: Attachment — Submitted filename: Response to reviewers.docx [file pone.0288571.s002.docx]

RESPONSE TO REVIEWERS

EDITOR IN CHIEF

PLOS ONE

The authors of the paper with new title “COMPARISON OF OPEN ANTERIOR COMPONENT SEPARATION AND OPEN TRANSVERSUS ABDOMINUS RELEASE IN REPAIR OF LARGE SUBCOSTAL HERNIAS” appreciate the reviewers for the observations made. All of them have been addressed and have greatly impacted on improving the overall quality of the document. Reposes are found belos.

1. Please ensure that your manuscript meets PLOS ONE's style requirements. Reviewer #1: Tile page Consider changing the topic to “COMPARISON OF OPEN ANTERIOR COMPONENT AND OPEN TRANSVERSUS ABDOMINUS RELEASE IN REPAIR OF LARGE SUBCOSTAL HERNIAS”

RESPONSE: The title of the study has been changed in the revised version of the manuscript.

ABSTRACT
Results
Paragraph 3, line 3: “undergoing” changed to “who had”
Paragraph 3, line 4: “undergoing” changed to “who had”
Conclusion
Paragraph 3, line 3: “undergoing” changed to “who had”
MATERIAL AND METHODS
Page 5, line 1: “who underwent” changed to “who had”
CONCLUSION
Page 9 line 2: The word “to” is changed to “with” before the phrase lower abdominal, the phrase “lower abdominal wall” is changed to “local” and the word “to” is changed to “with” before the phrase “lower recurrence”

RESPONSE: All these wordings been changed in the revised version of the manuscript.

2. 2. We note that you have indicated that data from this study are available upon request. PLOS only allows data to be available upon request if there are legal or ethical restrictions on sharing data publicly. For more information on unacceptable data access restrictions, please see http://journals.plos.org/plosone/s/data-availability#loc-unacceptable-data-access-restrictions.

In your revised cover letter, please address the following prompts:

a) If there are ethical or legal restrictions on sharing a de-identified data set, please explain them in detail (e.g., data contain potentially sensitive information, data are owned by a third-party organization, etc.) and who has imposed them (e.g., an ethics committee). Please also provide contact information for a data access committee, ethics committee, or other institutional body to which data requests may be sent.

b) If there are no restrictions, please upload the minimal anonymized data set necessary to replicate your study findings as either Supporting Information files or to a stable, public repository and provide us with the relevant URLs, DOIs, or accession numbers. For a list of acceptable repositories, please see http://journals.plos.org/plosone/s/data-availability#loc-recommended-repositories.

We will update your Data Availability statement on your behalf to reflect the information you provide.

RESPONSE: In the revised cover letter we have stated that there are no ethical nor legal restrictions on sharing a de-identified data set. Therefore, we have included a file with the data set that supports the information on the manuscript.

3. 3. PLOS requires an ORCID iD for the corresponding author in Editorial Manager on papers submitted after December 6th, 2016. Please ensure that you have an ORCID iD and that it is validated in Editorial Manager. To do this, go to ‘Update my Information’ (in the upper left-hand corner of the main menu), and click on the Fetch/Validate link next to the ORCID field. This will take you to the ORCID site and allow you to create a new iD or authenticate a pre-existing iD in Editorial Manager. Please see the following video for instructions on linking an ORCID iD to your Editorial Manager account: <https://www.youtube.com/watch?v=_xcclfuvtxQ>

RESPONSE: ORCID ID 0000-0002-6665-5507 has been included in the submission

4. 4. Please review your reference list to ensure that it is complete and correct. If you have cited papers that have been retracted, please include the rationale for doing so in the manuscript text, or remove these references and replace them with relevant current references. Any changes to the reference list should be mentioned in the rebuttal letter that accompanies your revised manuscript. If you need to cite a retracted article, indicate the article’s retracted status in the References list and also include a citation and full reference for the retraction notice.

RESPONSE: The reference list has been reviewed and no further changes have been made.

Antonio Espinosa-de-los-Monteros, M.D.

Chief of Plastic Surgery

Instituto Nacional de Ciencias Medicas y Nutricion

Mexico City. Mexico
